# *Pasteurella multocida* Pm0442 Affects Virulence Gene Expression and Targets TLR2 to Induce Inflammatory Responses

**DOI:** 10.3389/fmicb.2020.01972

**Published:** 2020-08-14

**Authors:** Fang He, Xiaobin Qin, Na Xu, Pan Li, Xiaoyan Wu, Lijie Duan, Yiyang Du, Rendong Fang, Philip R. Hardwidge, Nengzhang Li, Yuanyi Peng

**Affiliations:** ^1^College of Animal Science and Technology, Southwest University, Chongqing, China; ^2^College of Veterinary Medicine, Kansas State University, Manhattan, KS, United States

**Keywords:** *Pasteurella multocida*, *Pm0442*, virulence gene, TLR2, inflammatory responses

## Abstract

*Pasteurella multocida* is an important pathogenic bacterium of domestic animals. However, the mechanisms of infection are still poorly understood. Here, we found that *Pm0442* was dramatically up-regulated in infected mice among 67 predicted lipoproteins of *P. multocida* serotype A CQ2 strain (PmCQ2). To explore the role of *Pm0442* in virulence and the potential of the mutant as a vaccine, *Pm0442* mutant of PmCQ2 was successfully constructed. Then, the virulence characteristics, immune/inflammatory responses, and the survival rates of challenged mice were determined. As a result, it was found that the *Pm0442* deletion of PmCQ2 significantly decreased bacterial loads and inflammatory responses of lung tissue in mice, resulting in improved survival. Mechanically, *Pm0442* affects PmCQ2 capsular and lipopolysaccharide (LPS) synthesis and iron utilization-related genes expression affecting adhesion and phagocytosis. Furthermore, PM0442 bound directly to Toll-like receptor 2 (TLR2) to mediate the secretion of pro-inflammatory cytokine (IL-1β, TNF-α, IL-6, and IL-12p40) in macrophages via activation of the NF-κB, ERK1/2 and p38 signaling pathways. Notably, PmCQ2Δ0442 could provide 70–80% protection to mice challenged with 3.08 × 10^7^ CFU of PmCQ2. Our findings demonstrate that *Pm0442* is a virulence-related gene of PmCQ2, which provides new guidance for the prevention and control of Pasteurellosis.

## Introduction

*Pasteurella multocida* (*P. multocida*) is a Gram-negative bacterium that causes diseases in poultry, livestock, and humans, resulting in huge economic losses (Ryan and Feder, 2019). There are 5 capsular serotypes (A, B, D, E, and F), of which serotype A mainly causes respiratory-related diseases, such as pneumonia in cattle (Dabo et al., 2007). However, the interaction mechanism between bacteria and host is still unclear.

The virulence factors of *P. multocida* mainly include capsule, LPS, outer membrane proteins, iron utilization related proteins, fimbriae, and toxins (Peng et al., 2019). Lipoproteins contribute to the bacterial fitness and pathogenesis. For example, the lipoproteins PpmA and SlrA of *Streptococcus pneumoniae* present a very important role in efficient bacterial colonization of mice (Cron et al., 2009; Hermans et al., 2006); Lbp, a virulence factor of *Streptococcus pyogenes*, promotes the adhesion to epithelial cells (Terao et al., 2002). Lmb in *Streptococcus agalactiae*, mediates the adhesion to human laminin (Spellerberg et al., 1999). PM0442 is a lipoprotein located at outer membrane of *P. multocida* (Hatfaludi et al., 2010) that is significantly up-regulated during infection of mice (Li et al., 2016), suggesting *Pm442* may be a virulence related gene of *P. multocida*.

Toll-like receptors (TLRs) are capable of activating immune cells by recognizing the pathogen-associated molecular patterns (PAMPs) of bacteria, resulting in the expression and release of a variety of cytokines (Takeda and Akira, 2015). Lipoproteins can be recognized by TLR2 or other pattern recognition receptors of immune cells (Zahringer et al., 2008), leading to activation of downstream signaling pathways to induce inflammatory cytokines (Schenk et al., 2009; Kovacs-Simon et al., 2011). Recently, our study reported that TLR4 plays important roles in mediating the secretion of pro-inflammatory cytokines during PmCQ2 infection, but other TLRs might also be involved (Fang et al., 2019). Furthermore, our previous results showed that TLR2 was significantly up-regulated during PmCQ2 infection (Wu et al., 2017). Therefore, TLR2 may be another receptor that mediates the secretion of inflammatory cytokines in PmCQ2 infection.

In this study, we demonstrated that *Pm0442* deletion of PmCQ2 significantly improved mouse survival, decreased bacterial loads and inflammatory responses of mice lungs. Mechanically, *Pm0442* affects the expression of capsular, LPS, iron, and TonB receptor-related virulence genes. Furthermore, PM0442 induces the secretion of cytokines in macrophages though TLR2-NF-κB and MAPK signaling pathways. Additionally, PmCQ2Δ0442 provided 70–80% protection to mice challenged with PmCQ2 (3.08 × 10^7^ CFU). These results provide a new insight of infection mechanism to *P. multocida*.

## Materials and Methods

### Bacterial Strains and Growth Conditions

The highly virulent bovine *P. multocida* capsular type A CQ2 (PmCQ2) is isolated from a lung of calf with pneumonia in Chongqing, China (Du et al., 2017). PmCQ2 was streaked on Martin agar plate and incubated (Qingdao Hope Bio-Technology Co., Ltd., Qingdao, China) for 24 h at 37°C, and single colony was picked and inoculated to 5 mL Martin broth and cultured for 12 h at 37°C with shaking at 220 r/min.

### Experimental Animals and Ethics Statement

C57BL/6 and KM female mice (7–8 weeks old) were purchased from the Laboratory Animal Center of Third Military Medical University (Chongqing, China). C57BL/6 Tlr2^–/–^ and Tlr4^–/–^ mice were kindly provided by Dr. Feng Shao from NIBS (National Institute of Biological Sciences, Beijing). Total 136 C57BL/6 WT, 32 Tlr2^–/–^, 24 Tlr4^–/–^, and 140 KM mice were used in this study. The mice were housed in individually ventilated pathogen-free cages (temperature at 20–30°C, relative humidity at 50–60%, and lighting cycle at 12 h/day) with free access to food and water. The mice were acclimated for 4 days after arrival before starting the experiments. This study was carried out in accordance with the principles of the Basel Declaration and recommendations of the Laboratory Animal Welfare and Ethical Commission of the Southwest University (Permit No. 11-1025), Chongqing, China.

### Bioinformatics Analysis

Based on genome-wide sequence (GenBank accession number: CP033599.1) and transcriptome sequence (SRA accession: PRJNA597403) of *P. multocida* CQ2 strain, lipoproteins (Supplementary Table S1, S2) and iron absorption/utilization related proteins (Supplementary Table S3) of PmCQ2 were predicted according to the previously described method (Juncker et al., 2003).

### Mutant Construction

**FIGURE 1:**
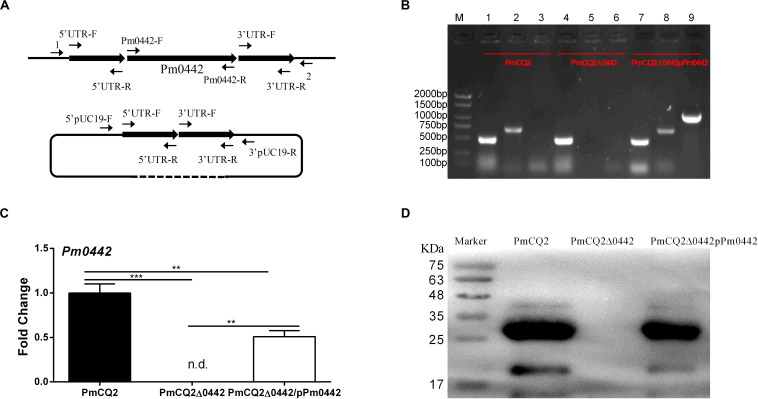
PmCQ2Δ0442 mutant construction. **(A)** Schematic to delete *Pm0442* gene by single-crossover recombination. **(B)** PCR confirmation of mutant and complementation strains. M: DNA marker; Lanes 1–3: PmCQ2; Lanes 4–6: PmCQ2Δ0442; Lanes 7–9: PmCQ2Δ0442/pPm0442. Lanes 1, 4, and 7: KMT1-F and KMT1-R (positive control); lanes 2, 5, and 8: Pm0442-F and Pm0442-R; Lanes 3, 6, and 9: Pm0442-F and pUC19-R. **(C,D)** *Pm0442* transcription and expression of were measured using qRT-PCR **(C)** and western blotting **(D)**. Panels **(B,D)** were pooled from three independent experiments. Panel **(C)** were pooled from three independent experiments with 6 replicates per group, and were analyzed by multiple comparative analysis, and expressed as means ± SD. **P < 0.01, ***P < 0.001.

To investigate whether *Pm0442* is related to the PmCQ2 virulence, we constructed a mutant of *Pm0442* by homologous recombination. Kanamycin (Kan) resistance gene expression cassettes combined with a fragment containing origin of DNA replication from temperature-sensitive (TS) plasmid pCP109 (Briggs and Tatum, 2005) were inserted between the endonuclease *Kpn*I and *Eco*RI of plasmid pUC19 to generate the replacement plasmid pUC19*ori*^TS^Kan^R^, which was able to replicate autonomously at 30°C, but not at 42°C. Then, the *Pm0442* gene expression cassettes with 690 bp *Pm0442*, 350-bp upstream segment and a 350-bp downstream segment were amplified with the primers Pm0442U-F/Pm0442U-R and Pm0442D-F/Pm0442D-R, respectively, and then linked by overlap PCR with Pm0442U-F/Pm0442D-R. Next, the PCR product was inserted into the *Hind*III and *Bam*HI sites of pUC19*ori*^TS^Kan^R^ to generate the plasmid pUC19*ori*^TS^KanR-Δ0442. Then, the plasmid was transferred into PmCQ2 by electroporation (voltage: 2,500 V, unit: 500 Ω, time: 5 ms). The cells were recovered at 30°C for 2 h and then spread onto Martin agar plates containing Kan (100 μg/mL) incubating for 24 h at 30°C. Individual colonies were transferred to 5 mL Martin broth with Kan (100 μg/mL) and incubated at 30°C for 24 h. 10 μL of broth from the transformed cell cultures was spread onto Martin agar plates with Kan (100 μg/mL) and grown at 42°C for 16 h to generate single-crossover mutants. Representative colonies were passed into 5 mL Martin broth lacking antibiotic and grown at 30°C for 16 h to allow resolution. The PmCQ2Δ0442 mutant was selected on Martin agar plates containing Kan (100 μg/mL) at 30°C. Subsequently, the candidate mutant clones were verified by PCR screening using primers Pm0442-F/Pm0442-R and pUC19-F/pUC19-R, as depicted in Figure 1A. As a positive control, the KMT1 gene was amplified with the primers KMT1-F/KMT1-R. The PmCQ2Δ0442 mutant was continuously passed in liquid Martin medium for 20 generations to verify the genetic stability. To complement the PmCQ2Δ0442 mutant, Kan resistance gene expression cassettes and a DNA replication origin sequence was inserted between the endonuclease *Kpn*I and *Eco*RI of plasmid pUC19 to generate the plasmid pUC19*ori*Kan^R^. Next, the *Pm0442* expression cassettes composed of a 162 bp upstream segment, a 45 bp downstream segment, and 690 bp *Pm0442* were amplified from PmCQ2 genomic DNA with the primers pPm0442U-F and pPm0442D-R. Then the amplified fragment was inserted into the *Hind*III and *Bam*HI sites of pUC19*ori*Kan^R^ to generate the complementary plasmid pUC19*ori*Kan^R^-Pm0442. Then the plasmids were transformed into the PmCQ2Δ0442 mutant strain, generating the corresponding complementary strain PmCQ2Δ0442/pPm0442. Primers sequences are listed in Table 1 and bacterial strains and plasmids are listed in Table 2.

**TABLE 1:** Mutant construction and protein expression primers used in this work.

**Primer name**	**Sequence (5′–3′)**	**Product (bp)**
Pm0442-F	GTGCCGCGCGGCAGCCATATG TGTTTTGATAAAGAGAGCAAAGC	690
Pm0442-R	GCAAGCTTGTCGACGGAGCTC TTATGCTTTAGGCACAATCACTTC	
Pm0442U-F	GACCATGATTACGCCAAGCTTA TCCTTCGACGATTGCGCAT	350
Pm0442U-R	TATCCATGCATTCTCCTAACTT TTACGAGG	
Pm0442D-F	GTTAGGAGAATGCATGGATAGA AATAGCGG	350
Pm0442D-R	TTTATCGGTACCCGGGGATCCA GCAAGTGATTTATGCCAAA	
KMT1-F	ATCCGCTATTTACCCAGTGG	460
KMT1-R	GCTGTAAACGAACTCGCCAC	
pUCI9-F	GAGCGGATAACAATTTCACAC	151
pUCI9-R	ATTTAAGAATACCTTGCCGC	
pPm0442-F	TTATCGGTACCCGGGGATCCTA GAATCATACTGTGGCGTT	897
pPm0442-R	ACCATGATTACGCCAAGCTTTA CCTCCGCTATTTCTATCC	
Pm0442-exp-F	GTGCCGCGCGGCAGCCATA TGTGTTTTGATAAAGAGAGCAAAGC	630
Pm0442-exp-R	GCAAGCTTGTCGACGGAGCT CTTATGCTTTAGGCACAATCACTTC	
Pm0442qPCR-F	TGGAAGAAGTGAAAGCCGCTACTG	135
Pm0442qPCR-R	CCATCTTTGTCGCCGTAGCAG	

**TABLE 2:** Bacterial strains and plasmids used in this study.

**Plasmids or strains**	**Description**	**Source**
**Plasmids**		
pET-28a	Basic plasmid for protein expression.	Lab collection
pET-28a-Pm0442	For the expression of *P. multocida* PM0442.	This work
pUC19	Basic plasmids for mutation and complement construction.	Lab collection
pUC19 *ori*^TS^Kan^R^	Insertion of Kan expression cassettes and a replication origin sequence with temperature sensitivity property into pUC19.	This work
pUC19*ori*^TS^Kan^R^ -ΔPm0442	For the deletion of *Pm0442* in *P. multocida* PmCQ2.	This work
pUC19*ori*Kan^R^	Insertion of Kan expression cassettes and a replication origin sequence of into pUC19.	This work
pUC19*ori*Kan^R^ -Pm0442	Insertion of Pm0442 into pUC19*ori*Kan^R^.	This work
**Strains**		
*E. coli* DH5α	For storing PM0442 expression plasmids pET-28a-Pm0442.	Lab collection
*E. coli* BL21	For the expression of PM0442 protein.	Lab collection
PmCQ2	Wild-type and virulent *P. multocida.*	Lab collection
PmCQ2-Δ0442	Pm0442 gene deleted strain in *P. multocida* PmCQ2.	This work
PmCQ2-Δ0442/pPm0442	Pm0442 gene complementary strain in PmCQ2-Δ0442.	This work

### Pathogenicity of Mutant Strain

To determine the pathogenicity of the PmCQ2Δ0442 mutant, C57BL/6 mice were infected by intranasal exposure with PmCQ2, PmCQ2Δ0442, or PmCQ2Δ0442/pPm0442 at a dose of 1 × 10^8^ CFU in 20 μL, and the numbers of mice used for each strain detection were equal. Mice were monitored for 7 days to determine the survival curves, and mice showing severe clinical signs (e.g., depression, accelerated breath, cough, hairiness, and lethargy) were considered moribund and were humanely killed. Survival rates (*n* = 12/group) were measured in three groups after injection. Mice were also euthanized for collection of lung tissues to measure the bacterial loads (*n* = 10/group), RT-PCR (*n* = 6/group), and ELISA (*n* = 8/group), and serum samples (*n* = 8/group) at 16 h post-infection for ELISA (He et al., 2019).

### 50% Lethal Dose (LD50) Measurements

To determine the impact of *Pm0442* on PmCQ2 virulence, 100 female KM mice (7/8-week-old) were randomly divided into 10 groups (*n* = 10/group). PmCQ2 experimental groups (5 groups) of mice were infected intramuscularly with 100 μL of various doses PmCQ2 (5.0 × 10^1^, 5.0 × 10^2^, 5.0 × 10^3^, 5.0 × 10^4^, and 5.0 × 10^5^ CFU). Experimental groups of PmCQ2Δ0442 (5 groups) were infected intramuscularly with 100 μL of various doses of PmCQ2Δ0442, ranging from 2.0 × 10^5^ to 2.0 × 10^9^ CFU. For all groups, mice were monitored for 7 days to determine the survival curves, and mice showing severe clinical signs were considered moribund and were humanely euthanized. Then the numbers of surviving mice in each group were recorded, and the LD50 was calculated with the method of Bliss (Liu L. et al., 2017).

### Histopathological Examination (HE) Staining of Mice Lung Tissues

To explore the effect of PmCQ2Δ0442 on the inflammation of lung tissues in mice, the HE experiments were performed as described in the previous study (He et al., 2019). The lung tissues (*n* = 6/group) were immediately fixed in the 4% paraformaldehyde for 24 h, dehydrated in graded ethanol, and then embedded in paraffin wax. The tissues were sliced at 3 μm thick and then stained with hematoxylin and eosin (H&E). Histopathological scoring was performed by a pathologist blinded to treatment and control groups, and the scoring was mainly based on interstitial inflammation, vascular endothelialitis, bronchitis, edema, serous effusion and thrombus formation, all parameters were scored separately from 0 (lesion absent) to 3 (severe lesion) (Wu et al., 2017).

### Immunization and Challenge Trial

To determine immune protection of PmCQ2Δ0442, 40 female KM mice were randomly divided into four groups (*n* = 10/group). Three experimental groups of mice were intramuscularly inoculated with 100 μL of different concentrations of PmCQ2Δ0442 (2.88 × 10^4^, 2.88 × 10^5^, and 2.88 × 10^6^ CFU), and the control group mice were inoculated with 100 μL PBS. The mice were intramuscularly infected with 3.08 × 10^7^ CFU PmCQ2 at day-21 after inoculation. The mice were monitored for 10 days after challenge, which showing severe clinical signs were considered moribund and were humanely euthanized, and the numbers of surviving mice in each group were recorded.

### Transcriptome Analysis

To explore the details of PmCQ2Δ0442 virulence decrease, C57BL/6 mice were infected by an intranasal inoculation with PmCQ2 (*n* = 3) and PmCQ2Δ0442 (*n* = 3) at the dose of 1 × 10^8^ CFU in 20 μL. Mice were euthanized at 16 h post-infection, lung tissues were collected and quickly frozen in liquid nitrogen. Simultaneously, one milliliter fresh mid-logarithmic phase of PmCQ2 (*n* = 3) and PmCQ2Δ0442 (*n* = 3) were seeded in 100 mL fresh Martin liquid medium and incubated at 37°C with shaking at 220 r/min for 12 h. Bacterial cultures were centrifuged at 3,000 r/min for 10 min at 4°C, the cell pellets were suspended in 10 mL ice-cold PBS and centrifuged again. The bacterial cells were quickly frozen in liquid nitrogen after washed three times. Lung tissues and bacteria samples were sent to the Beijing Genomics Institute (BGI, Shenzhen, China) for transcriptome sequencing and analysis (HiSeq, Illumina, United States). The data in this study have been deposited to NCBI’s Sequence Read Archive (SRA) database and the accession number is PRJNA597831.

### Transmission Electron Microscopy (TEM)

To detect the capsule thickness of the PmCQ2Δ0442, bacterial samples were prepared for TEM as described previously (Fang et al., 2020). Briefly, 1 mL fresh mid-logarithmic phase of PmCQ2, PmCQ2Δ0442, and PmCQ2Δ0442/pPm0442 were seeded in 100 mL fresh Martin liquid medium and incubated at 37°C with 220 r/min for 8 h. Bacterial cultures were centrifuged at 3,000 r/min for 10 min at 4°C, the cell pellets were suspended in 10 mL ice-cold PBS and centrifuged, repeated three times. Cells were fixed with 2.5% fresh glutaraldehyde for 6 h and gently washed three times with 10 mL PBS for 10 min each time, then treated with 1–2% citric acid for 2–3 h and gently washed three times with PBS for 10 min each time. Fixed samples were dehydrated gradually with ethanol (30, 50, 70, 80, 90, and 100%) and then with acetone. Bacterial samples were placed on copper mesh grids with formvar membranes and negatively stained with phosphotungstic acid (2% v/v, pH = 6.7). The samples were then observed using a TEM (Hitachi H-7650) at 80 kV and photographed with a Gatan832 CCD camera (Gatan, Pleasanton, CA, United States). Each experiment was performed two times with three replicates.

### Quantification of Hyaluronic Acid in the Capsule of *P. multocida*

To determine the hyaluronic acids content of PmCQ2Δ0442, bacteria were grown in 100 mL fresh Martin liquid medium and incubated at 37°C with 220 r/min for 8 h. Then the culture was centrifuged at 7,600 *g* for 15 min and the supernatant was removed. Next, the bacterial cells were washed twice with PBS. The bacteria were then re-suspended in PBS and incubated for 1 h at 42°C. At the same time, the number of bacteria was counted on Martin agar plates before and after incubation at 42°C. Bacterial solutions were centrifuged and the supernatant was collected for the detection of capsule content as previously described method (Fang et al., 2020). Briefly, 10 μL sample and 10 μL hyaluronic acid standards were added to 90 μL capsule staining solution (0.2 g/mL Stain all staining solution, 0.06% glacial acetic acid in 50% formamide). Then, absorption of OD640 was determined by microplate reader and capsule content was calculated.

### LPS Quantification

To determine the LPS content of PmCQ2Δ0442, bacteria were grown in fresh Martin liquid medium and incubated at 37°C with 220 r/min for 8 h. Next, the bacterial cells were washed twice with PBS. Bacterial cultures were centrifuged at 3,000 r/min for 10 min at 4°C, then the supernatants were collected and the cell pellets were washed three times by 10 mL ice-cold PBS. Bacteria were then lysed with ultrasound for 5 min, working for 1 s, interval of 3 s (150 W). Then the LPS content in bacterial culture and bacterial lysate were determined using an ELISA Kit (Cloud-Clone Crop., Houston, TX, United States), which is used for the quantification of LPS.

### Peritoneal Macrophage Isolation, Culture and Bacterial Infection

Peritoneal macrophages were obtained according to previous methods (Fang et al., 2019). Briefly, C57BL/6 WT, Tlr2^–/–^, and Tlr4^–/–^ mice were injected intraperitoneally (i.p.) with 2mL 4% thioglycolate broth (Eiken, Japan), and peritoneal macrophages were collected 3 days later. The peritoneal macrophages were suspended in RPMI 1640 medium (Gibco, United States) containing 10% FBS (Gibco, United States) and counted with a hemocytometer. The cells were seeded in 24-well microplates at a density of 5 × 10^5^ cells/well and incubated at 37°C in 5% CO_2_. The non-adherent cells were removed after 2 h. Then adherent cells were challenged with PmCQ2, PmCQ2Δ0442 and PmCQ2Δ0442/pPm0442 at a multiplicity of infection (MOI) of 1 for 24 h. The supernatants were collected for cytokines detection by ELISA.

### Quantitation of *P. multocida* Associated With Macrophage

According to the previously described method (Fang et al., 2019, 2020; He et al., 2019), briefly, the adherent cells were cultured in 48-well microplates (2 × 10^5^ cells/well) with RPMI 1640 medium and challenged with *P. multocida* at an MOI of 1 for 3 h. Cells were washed three times with 4°C chilled PBS to remove non-associated bacteria and lysed in PBS containing 0.1% Triton X-100. Then the cell lysates were diluted with PBS and grown on Martin’s agar plates at 37°C for 18–24 h to determine the number of *P. multocida* associated with macrophage (total number of adhered and phagocytosed *P. multocida*).

### Expression and Purification of rPM0442 Protein

To explore the function of PM0442 protein, the *pm0442* gene was amplified and inserted into pET28a plasmids and induced for expression in *Escherichia coli* BL21(DE3). Purified recombinant PM0442 (rPM0442) proteins were prepared in accordance with previously described protocols (Du et al., 2017), and desalted via Zeba^TM^ Spin Desalting Columns, 7K MWCO (Thermo, Germany). Then endotoxin was removed by using Toxin Eraser^TM^ endotoxin removal kit (Genscript, United States) and tested through ToxinSensorTM Chromogenic LAL Endotoxin Assay Kit (Genscript, United States).

### PM0442 Function Assay

To investigate whether PM0442 protein is involved in the secretion of inflammatory cytokines from macrophages. Endotoxin-free rPM0442 proteins (5, 10, and 15 μg) and the parallel controlling factors including LPS (1 μg/mL, Sigma), Boiled rPM0442 (15 μg/mL, incubation at 100°C for 20 min) and Proteinase K (PK) + rPM0442 (50 μg/mL + 15 μg/mL) were added to the cell culture plate wells containing peritoneal macrophages (5 × 10^5^ cells/well), respectively, and the blank control (UI) macrophages received medium only. Then the peritoneal macrophages were cultured at 37°C, 5% CO_2_ for 24 h, and the supernatants and pellets were collected after centrifugation at 5,000 r/min for 5 min, the supernatants were used for the detection of secreted cytokines (IL-1β, TNF-α, IL-6, and IL-12p40), and cell pellets were used for RNA extraction to detect the expression of TLRs by qRT-PCR.

### Quantitative Real-Time-PCR (qRT-PCR)

All RNA extractions were performed using an RNAprep pure Animal/Cell/Bacteria Kit (TIANGEN, Beijing, China) involving an gDNA elimination step. cDNAs were synthesized with an iScript cDNA synthesis kit (Bio-Rad, CA, United States), and quantitative real-time RT-PCR was performed according to previous study using a CFX96 instrument (Bio-Rad, CA, United States) (Wu et al., 2017). qRT-PCR Primers sequences are listed in Supplementary Table S4.

### Measurement of MAPK and NF-κB Signaling Pathways by Western Blot Analysis

Peritoneal macrophages were cultured in 12-well plates at a density of 1 × 10^6^ cells/well in RPMI 1640 containing 10% FBS at 37°C for 2 h. After washing with PBS three times, the culture medium was replaced by RPMI 1640 without FBS, and the adherent cells were incubated with 10 μg rPM0442 protein. The cells were collected after 0, 5, 15, 30, and 60 min of incubation, and were lysed with radio-immunoprecipitation assay (RIPA) buffer (Beyotime, China). The cell lysates were subjected to SDS-PAGE and subsequently transferred to 0.2 μm polyvinylidene difluoride (PVDF) membranes by electroblotting. The membranes were immunoblotted with antibodies specific to IκBα, p-IκBα, NF-κB, p38, p-p38, ERK (1/2), p-ERK (1/2), JNK, p-JNK (Cell Signaling Technology, United States) and β-actin (Proteintech, China).

### Antibody Blocking Experiment

To further verify that rPM0442 protein induces the secretion of inflammatory cytokines in macrophages through MAPK and NF-κB signaling pathways. 10 μg endotoxin-free rPM0442 protein in RPMI 1640 medium containing 10% FBS was incubated with peritoneal macrophages washed three times with PBS. The culture supernatants were collected and used after 24 h of incubation. Inhibitors of NF-κB (BAY11-7082), JNK (SP600125), p38MAPK (SB203580), and ERK (U0126) were purchased from Beyotime (Shanghai, China), TLR2Ab from Thermo (United States), TLR4Ab from Santa (United States). All inhibitors or specific antibody were added to cultures 1 h before rPM0442 protein incubation. Levels of secreted cytokines in the culture supernatants were determined by ELISA. ELISA kits for IL-1β, TNF-α, IL-12p40, IL-6 were purchased from eBioscience (San Diego, CA, United States).

### His-Pull-Down Assay

To investigate whether PM0442 protein directly binds to TLR2, the purified His-rPM0442 (500 μg) proteins were mixed with 100 μL of Ni-NTA (His-tag affinity) magnetic beads and gently mixed, parallel control was only containing 100 μL of Ni-NTA magnetic beads. The mixture was placed on a rotator and slowly mixed for 30 min, carefully removed the liquid on the magnetic stand. Next, the mixture was mixed with 500 μL peritoneal macrophage lysate and incubated overnight at 4°C on a rotator. Then the liquid was removed and washed three times with 4°C precooled PBS on a magnetic stand. Finally, it was re-suspended in 100 μL precooled PBS, which was the final sample of pull-down. The samples were subjected to SDS-PAGE and then detected using anti-His (Thermo, Germany) and anti-TLR2 antibodies (Thermo, Germany) with macrophage lysate and rPM0442 as control.

### Statistical Analysis

Data were shown as the means ± standard deviation (SD). Data between two groups were analyzed using unpaired t tests (Prism 6.0) if the data were in Gaussian distribution and had equal variance, or by unpaired *t*-test with Welch’s correction (Prism 6.0) if the data are in Gaussian distribution but show unequal variance, or by non-parametric test (Mann–Whitney *U* test, Prism 6.0) if the data were not normally distributed. The Gaussian distribution of data was analyzed by D’Agootino-Pearson omnibus normality test (Prism 6.0) and Kolmogorov–Smirnov test (Prism 6.0). The variance of data was analyzed by Brown–Forsythe test (Prism 6.0). Differences with *p* < 0.05 were considered significant.

## Results

### Construction of the PmCQ2Δ0442 Mutant

Our previous findings indicated that *Pm0442* was highly expressed in mice infected by PmCQ2 (Li et al., 2016), thus, we speculated *Pm0442* may be a potential virulence gene. The *Pm0442* gene was deleted from PmCQ2 using method of temperature sensitive plasmid-mediated homologous recombination (Figure 1A) and verified using KMT1-F/R and Pm0442-F/R gene detection. The *Pm0442* gene (Pm0442-F and Pm0442-R) was present in parent strain PmCQ2 and complemented strain PmCQ2Δ0442/pP0442, but not in the mutant strain PmCQ2Δ0442, and the positive control KMT1 could be amplified from three strains (Figure 1B). Transcription and expression of *Pm0442* were also measured using qRT-PCR and WB (polyclonal anti-PM0442 antibody), respectively. *Pm0442* was transcribed and expressed in the parent strain PmCQ2 and the complementary strain PmCQ2Δ0442/pP0442, but not in the mutant strain PmCQ2Δ0442 (Figures 1C,D), demonstrating that the *Pm0442* gene was successfully deleted in PmCQ2. Moreover, PmCQ2Δ0442 was stable for more than 20 passages (data not shown).

### The Phenotype of the PmCQ2Δ0442 Mutant

**FIGURE 2:**
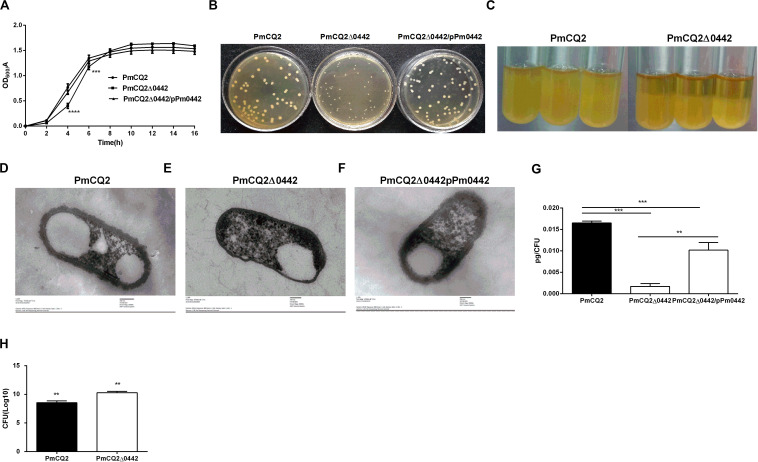
PmCQ2Δ0442 characteristics. **(A)** Bacterial growth curves based on OD_600_ nm reading. **(B)** Colony morphology. **(C)** PmCQ2Δ0442 and PmCQ2 strains after standing for the same time. **(D–F)** Capsule thickness of fresh cultured PmCQ2 **(D)**, PmCQ2Δ0442 **(E)** and PmCQ2Δ0442/pPm0442 **(F)** by TEM. **(G)** The content of capsule polysaccharide in PmCQ2, PmCQ2Δ0442, and PmCQ2Δ0442/pPm0442. **(H)** The number of PmCQ2Δ0442 associated with macrophages. **(A,B)** were pooled from three independent experiments with three replicates per group. Panels **(G,H)** were pooled from two independent experiments with eight replicates per group. And **(H)** were determined by multiple comparative analysis, **(G)** was analyzed by unpaired *t*-test. All data were expressed as means ± SD. (**P < 0.01, ***P < 0.001, ****p < 0.0001).

The parent strain PmCQ2 showed a typical growth curve, with a short lag phase (0–2 h), followed by a log phase during which major bacterial growth occurred (2–10 h) and then a stationary phase (10–14 h). In contrast, PmCQ2Δ0442 grew more slowly at 0–4 h, but the growth rate was reversed at 4–10 h. The OD values of PmCQ2Δ0442 were significantly lower than those of the parent strains between 0 and 8 h. The complementary strain PmCQ2Δ0442/pP0442 partially restored the defective growth, but the OD values remained lower than those of the parent strain (Figure 2A). The colony morphology of PmCQ2Δ0442 colonies was significantly smaller than that of PmCQ2 and PmCQ2Δ0442/pP0442 and had a slower growth rate (Figure 2B). At the same time, PmCQ2Δ0442 was easier to centrifuge and precipitate than PmCQ2 (Figure 2C). Furthermore, the thickness and content of capsule in PmCQ2Δ0442 were decreased (Figures 2D,E,G), and PmCQ2Δ0442/pP0442 partly restored capsule (Figures 2F,G). The numbers of PmCQ2Δ0442 associated with macrophages were increased (Figure 2H). The above results indicate that *Pm0442* gene may affect the formation of PmCQ2 capsule.

### Role of *Pm0442* in *P. multocida* Virulence

**FIGURE 3:**
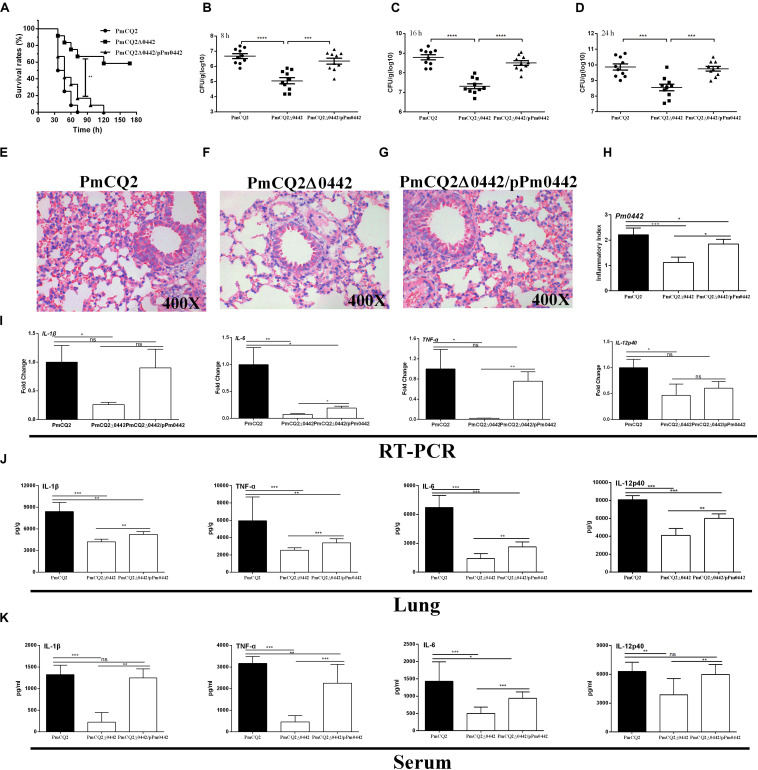
PmCQ2Δ0442 virulence. **(A)** Survival rates of mice as a function of time after infection. **(B–D)** Bacterial burdens in the lungs at 8, 16, and 24 h post-infection. **(E–H)** H&E staining of mouse lungs at 16 h post infection. **(I–K)** The mRNA expression **(I)** and the protein abundance of IL-1β, IL17, IFN-γ, and TNF-α in the lung **(J)** and serum **(K)** of mice at 16 h post-infection. Panels **(A–D)** were pooled from three independent experiments with 10 replicates per group. Panels **(E–I)** were pooled from two independent experiments with 6 replicates per group. Panels **(J,K)** were pooled from two independent experiments with 8 replicates per group. Panel **(A)** was were determined by Log-rank (Mantel–Cox) test. Panels **(B–D)** were analyzed with unpaired *t*-test. Panels **(H–K)** were determined by one-way ANOVA, unpaired *t*-test or Mann–Whitney test. The data were expressed as means SD (*p < 0.05, **p < 0.01, ***p < 0.001, ****p < 0.0001).

The LD50 of PmCQ2Δ0442 was 5.96 × 10^8^ CFU, which was ∼174,000-fold higher than that of the parent strain PmCQ2, whose LD50 was 3.43 × 10^3^ CFU (Table 3). After infection by PmCQ2, PmCQ2Δ0442 and PmCQ2Δ0442/pP0442 by intranasal injection (1 × 10^8^ CFU), the survival rates (58.3%) of mice infected with PmCQ2Δ0442 was significantly increased (Figure 3A), and the bacteria colonization were significantly decreased in the mouse lungs at 8, 16, and 24 h post-infection (Figures 3B–D). PmCQ2Δ0442 also induced a lower inflammatory responses based on the H&E staining in the lung (Figures 3E–H). Compared with the PmCQ2-infected group, the PmCQ2Δ0442-infected group showed significant reduction in the levels of IL-1β, TNF-α, IL-6, and IL-12p40 of mice lung and serum (Figures 3I–K).

**TABLE 3:** Determination of the LD50 in PmCQ2 and PmCQ2Δ0442.

PmCQ2	Infection dose (CFU)	5.0 × 10^5^	5.0 × 10^4^	5.0 × 10^3^	5.0 × 10^2^	5.0 × 10^1^
	Survive/total mice	0/10	4/10	6/10	7/10	7/10
LD_50_ = 3.43 × 10^3^CFU						

PmCQ2Δ0442	Infection dose (CFU)	2.0 × 10^9^	2.0 × 10^8^	2.0 × 10^7^	2.0 × 10^6^	2.0 × 10^5^
	Survive/total mice	3/10	7/10	9/10	10/10	10/10
LD_50_ = 5.96 × 10^8^CFU						

### PmCQ2Δ0442 Is a Potential Vaccine Candidate Strain

To evaluate the protective efficacy of PmCQ2Δ0442, 6-week-old mice were immunized with different doses of PmCQ2Δ0442 (2.88 × 10^4^, 2.88 × 10^5^, and 2.88 × 10^6^ CFU) by intramuscular injection. Mice were challenged with approximately 3.08 × 10^7^ CFU PmCQ2 at day 21 after the immunization. In the immunized group, 70–80% of the mice survived after challenge, while all control mice were dead within 1 week (Table 4), indicating that the PmCQ2Δ0442 is a potential vaccine candidate that could provide moderate protection against *P. multocida* infection.

**TABLE 4:** Evaluation of the protection rate conferred by PmCQ2-Δ0442 mutant.

**Group**	**Immunization**	**Challenge**	**Survival**	**Protection**
Immunization group1	2.88 × 10^4^ PmCQ2-Δ0442	3.08 × 10^7^ PmCQ2	7/10	70%
Immunization group2	2.88 × 10^5^ PmCQ2-Δ0442	3.08 × 10^7^ PmCQ2	8/10	80%
Immunization group3	2.88 × 10^6^ PmCQ2-Δ0442	3.08 × 10^7^ PmCQ2	7/10	70%
Control	PBS	3.08 × 10^7^ PmCQ2	0/10	0

### *Pm0442* Affects Expression of Other Virulence Genes in PmCQ2

**FIGURE 4:**
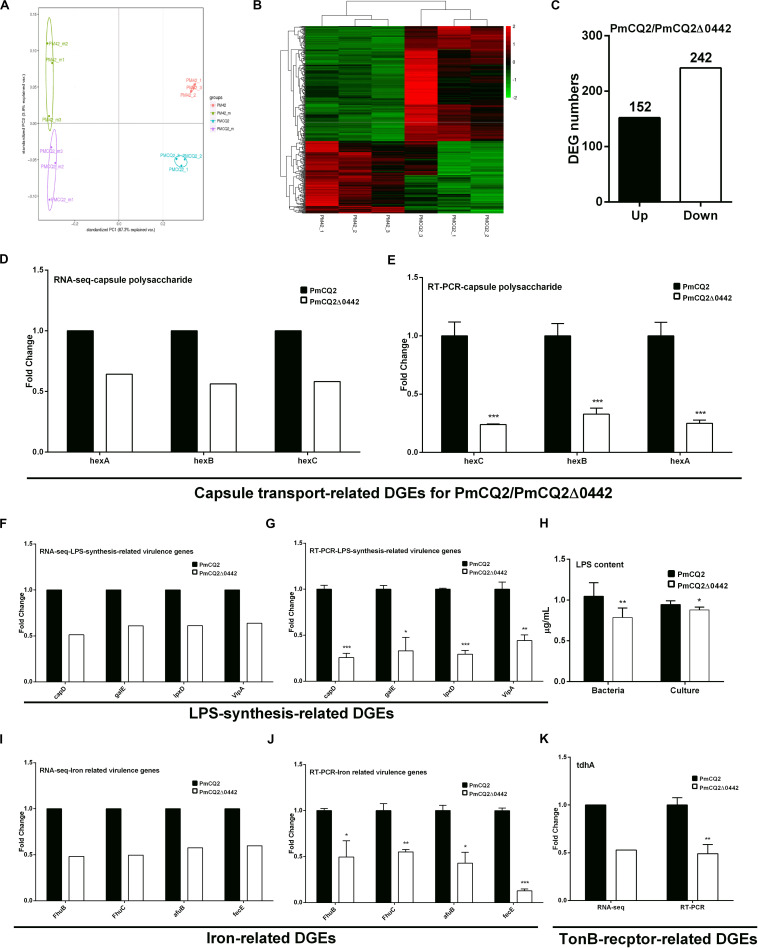
Virulence gene transcription *in vitro*. **(A)** Principal component analysis (PCA) of PmCQ2 and PmCQ2Δ0442 *in vitro* and *in vivo* (*n* = 3). **(B)** Heat map for clustering of differentially expressed genes (DEGs) (FC ≥ 1.5) of PmCQ2 and PmCQ2Δ0442 *in vitro*. **(C)** The up/down-regulated DEGs (FC ≥ 1.5) of PmCQ2 and PmCQ2Δ0442 *in vitro*. **(D,E)** Capsular polysaccharide transport-related DEGs (FC ≥ 1.5) in RNA-seq **(D)** and in RT-PCR (*n* = 3) **(E)**. **(F,G)** LPS synthesis related DEGs (FC ≥ 1.5) in RNA-seq **(F)** and in RT-PCR (*n* = 3) **(G)**. **(H)** Determination of LPS content in PmCQ2, PmCQ2Δ0442 and their culture. **(I,J)** Iron-related DEGs (FC ≥ 1.5) in RNA-seq **(I)** and in RT-PCR (*n* = 3) **(J)**. **(K)** TonB-receptor-related DEGs (FC ≥ 1.5) in RNA-seq and in RT-PCR (*n* = 3). Panels **(E,G,J,K)** were pooled from three independent experiments with 3 replicates per group and analyzed with multiple comparative analysis. Panel **(H)** was pooled from two independent experiments with 8 replicates per group and analyzed by unpaired *t*-test. All data were expressed as means ± SD (*p < 0.05, **P < 0.01, ***P < 0.001).

**FIGURE 5:**
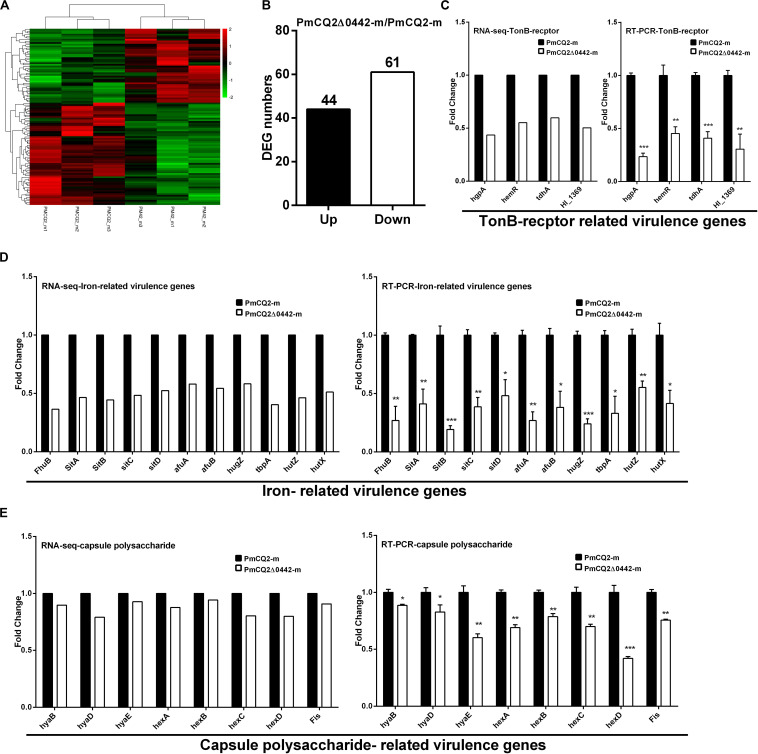
Virulence gene transcription *in vivo*. **(A)** Heat map for clustering of DEGs (FC ≥ 1.5) of PmCQ2 and PmCQ2-Δ0442 *in vivo*. **(B)** The up/down-regulated DEGs (FC ≥ 1.5) of PmCQ2 and PmCQ2-Δ0442 *in vivo*. **(C)** TonB receptor-related DEGs (FC ≥ 1.5) in RNA-seq and in RT-PCR (*n* = 3). **(D)** Iron-related DEGs (FC ≥ 1.5) in RNA-seq and in RT-PCR (*n* = 3). **(E)** Capsule synthesis and transport- related DEGs (FC ≥ 1.5) in RNA-seq and in RT-PCR (*n* = 3). Panels **(C–E)** were representative of two independent experiments with 3 replicates per group and analyzed by multiple comparative analysis, and expressed as means ± SD (*p < 0.05, **p < 0.01, ***p < 0.001).

To explore the virulence mechanism of *Pm0442*, we compared the differentially expressed genes (DEGs) of PmCQ2 and PmCQ2Δ0442 by transcriptome analysis *in vitro* and *in vivo*. A total of 394 DEGs were observed, including 152 up-regulated and 242 down-regulated genes *in vitro* (Fold change ≥ 1.5) (Figures 4A–C). GO and KEGG pathway analysis demonstrated that the top 93 DEGs were mainly involved in membrane transport, carbohydrate and energy metabolism, and amino acids synthesis, and these DEGs from RNA-sequence analysis were also validated by RT-PCR analysis (Supplementary Figure S1). Capsule synthesis and transport (Figures 4D,E and Supplementary Figure S2), and LPS synthesis (Figures 4F,G), iron utilization/transport (Figures 4I,J) and TonB-dependent receptor (Figure 4K) related virulence genes were significantly down-regulated in PmCQ2Δ0442 *in vitro*. Consistently, the content of LPS were decreased (Figure 4H). Similarly, a total of 105 DEGs were observed *in vivo*, including 44 up-regulated and 61 down-regulated genes (Fold change ≥ 1.5) (Figures 5A,B). TonB-dependent receptor and iron utilization/transport, and capsule synthesis/transport related DGEs significantly reduced (Figures 5C–E). The above results indicate that *Pm0442* affects virulence by affecting the capsules, LPS, and utilization of iron.

### PM0442 of *P. multocida* Targets TLR2 to Induce the Secretion of Inflammatory Cytokines in Macrophages

**FIGURE 6:**
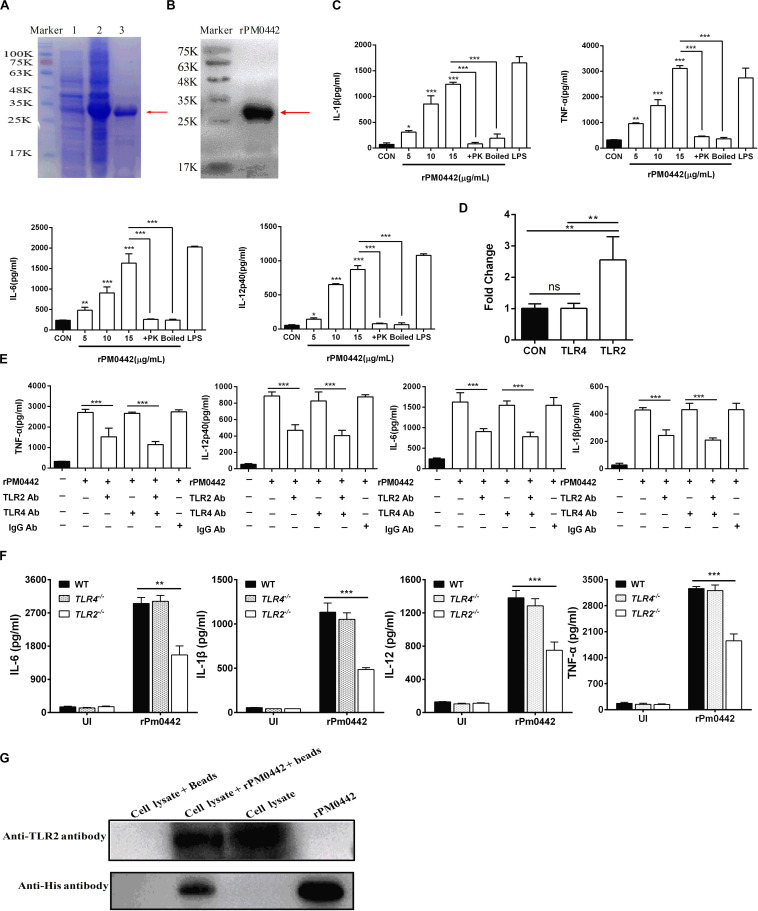
PM0442 promotes cytokine secretion by interacting with TLR2. **(A,B)** SDS-PAGE **(A)** and western blot images **(B)** for expression and purification of rPM0442 protein of *P. multocida*. Lane 1: The untranslated protein band, Lane 2: the expression of the rPM0442 protein band induced by IPTG, 3: the protein band after purification by the HIS-Ni column. **(C)** rPM0442 induces secretion of IL-1β, TNF-α, IL-6, and IL-12p40 in peritoneal macrophages. **(D)** TLR2 and TLR4 expression after rPM0442 protein treatment. **(E)** TLR2-specific antibodies reduce the production of IL-1β, TNF-α, IL-6, and IL-12p40 in macrophage induced rPM0442 protein. **(F)** TLR2^– /–^ macrophages have reduced secretion of IL-1β, TNF-α, IL-6, and IL-12p40 in macrophage induced rPM0442 protein. **(G)** Pull-down experiment to analyze the binding of rPM0442 to TLR2. Panels **(A,B,G)** were representative of three independent experiments. Panels **(C–F)** representative of three independent experiments with 8 replicates per group. Panel **(C)** was determined by one-way ANOVA. Panels **(D–F)** were analyzed with by unpaired *t*-test or Mann–Whitney test. The data significance was expressed as means ± SD (*P < 0.05, **P < 0.01, ***P < 0.001).

Bioinformatics analysis found that PmCQ2 has 67 lipoproteins (Supplementary Table S1). Notably, transcriptome sequencing found that 37 of them were significantly up-regulated during infection, including PM0442 (Supplementary Table S2). Lipoproteins are PAMPs of TLR2 (Pecora et al., 2006), thus, we speculated that PM0442 may be an agonist of TLR2. Purified rPM0442 protein (Figures 6A,B) was incubated with macrophages and induced dose-dependent secretion of IL-1β, TNF-α, IL-6, and IL-12p40 in both peritoneal macrophages (Figure 6C) and RAW264.7 macrophages (Supplementary Figure S3). At the transcriptional level, the rPM0442 protein induced TLR2, but not TLR4 expression (Figure 6D). TLR2-blocking antibodies, but not TLR4-blocking antibodies reduced the secretion of cytokines from macrophages treated with rPM0442 (Figure 6E). The secretion of cytokines after rPM0442 treatment was reduced in TLR2^–/–^ macrophages, but not TLR4^–/–^ macrophages (Figure 6F). Cytokine secretion from macrophages infected with PmCQ2Δ0442 was also reduced as compared with macrophages infected with wild-type PmCQ2 (Supplementary Figure S4, S5). rPM0442 protein appeared to interact with TLR2 in macrophages as assessed using His-pull-down experiments (Figure 6G).

### PM0442 Induces Secretion of Cytokines in Macrophages Dependent on NF-κB and MAPKs

**FIGURE 7:**
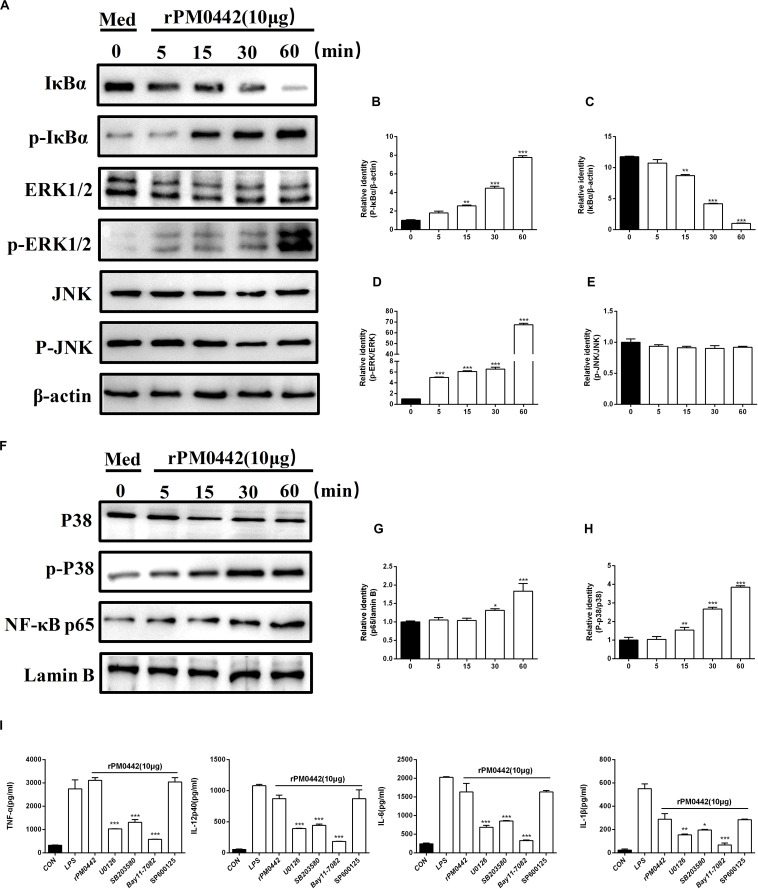
PM0442 induces cytokine secretion dependent on NF-kB and MAPKs signaling pathways. **(A–H)** Peritoneal macrophage incubated with 10 μg/mL rPM0442 protein for 0, 15, 30, or 60 min. Then, the cell lysates were collected, and the content of IκBα, ERK, and JNK in cytoplasm and NF-κB (p65) and P38 in nuclear, and total phosphorylated IκBα, ERK and JNK levels were detected by western blotting with β-actin (in cytoplasm) and Lamin B (in nuclear) as a control. **(I)** Peritoneal macrophages incubated with 10 μg/mL rPM0442 protein for 24 h, and then the supernatants were collected. The amounts of IL-1β, TNF-α, IL-6, and IL-12p40 in the supernatants were determined by ELISA. All inhibitors were added to cultures 1 h before rPM0442 protein incubation. The targets and concentrations of the kinase inhibitors are as follows: U0126 (ERK inhibitor, 10 μM), SB203580 (p38 inhibitor, 30 μM), BAY11-7082 (NF-κB inhibitor, 20 μM) and SP600125 (JNK inhibitor, 10 μM). Panels **(A–H)** were representative of two independent experiments with 4 replicates per group. Panel **(I)** was pooled from two independent experiments with 6 replicates per group. Panels **(B–E,G–I)** were determined by one-way ANOVA and expressed as means ± SD (*P < 0.05, **P < 0.01, ***P < 0.001).

To determine whether PM0442 protein activates NF-κB and MAPKs pathways in macrophages, 10 μg rPM0442 was used to treat macrophages, and the activation of these pathways were analyzed. The amount of p-IκBα increased with time, while the amount of IκBα in the cytoplasm decreased over time (Figures 7A–C). At the same time, the amount of nuclear NF-κB (p65) increased with time (Figures 7F,G). rPM0442 protein rapidly activated p38 (Figures 7F,H) and ERK1/2 (Figures 7A,D) in a time-dependent manner, but had no effect on JNK (Figures 7A,E). This indicates that PM0442 protein activate the NF-κB, p38 and ERK1/2 pathways in macrophages. To further determine the roles of NF-κB and MAPK signaling pathways in the secretion of cytokines induced by PM0442 protein, specific inhibitors of both pathways were used. NF-κB inhibitor BAY11-7082, p38 inhibitor SB203580 and ERK inhibitor U0126, but not JNK inhibitor SP600125, strongly inhibited rPM0442 protein-induced macrophage secretion of IL-1β, TNF-α, IL-6, and IL-12p40 (Figure 7I). These results indicate that PM0442 protein induces macrophage secretion of IL-1β, TNF-α, IL-6, and IL-12p40 dependent on NF-κB, p38, and ERK1/2 signaling pathways.

## Discussion

*Pasteurella multocida* is associated with a wide range of diseases in poultry, livestock and even humans. Virulence factors contribute to pathogenesis of *P. multocida*, including capsule, LPS, iron-regulated/acquisition proteins, OMPs, fimbriae, and toxins (PMT) (Harper et al., 2006). There are 5 capsular serotypes (A, B, D, E, and F) in *P. multocida*, the capsules of A, B, D, and F *P. multocida* are polysaccharides (Harper et al., 2012), but the composition of the capsule in *P. multocida* serotype E remains unclear. The A-type capsules are mainly composed of hyaluronic acid, heparin and chondroitin components (Boyce et al., 2000). The important roles of capsule in the pathogenesis of *P. multocida* have been clearly demonstrated, as the virulence of acapsular mutants constructed from different serogroups was strongly attenuated in mice (Boyce and Adler, 2000; Chung et al., 2001). Here we found that the PmCQ2Δ0442 mutant was strongly attenuated compared to the wild-type strain, and the colony morphology of PmCQ2Δ0442 mutation was significantly smaller than that of PmCQ2 and had a slower growth rate at 0–4 h. Moreover, PmCQ2Δ0442 was easier to centrifuge and precipitate compared to the PmCQ2. Additionally, it was found that the thickness and total capsule content was significantly reduced in PmCQ2Δ0442. Expression of the capsule in *P. multocida* type A requires 10 genes, including *phyAB* (phospholipid substitution), *hyaBCDE* (capsule biosynthesis), and *hexABCD* (capsule export) (Townsend et al., 2001). RNA sequencing showed that the genes related to capsule synthesis/transport were significantly down-regulated after deleting *pm0442*. The currently known proteins that regulate capsule synthesis are Fis and Hfq protein. Hfq is an RNA-binding protein, and the transcription level of the capsule-related genes of its mutants is also significantly reduced (Mégroz et al., 2016). Fis protein combined with the promoter sequence of the *hyaE* gene can increase the transcription of the capsular related genes (Steen et al., 2010). However, the mechanism of *Pm0442* affecting capsule synthesis still needs further experimental verification.

Lipopolysaccharide is also recognized as one of the most important factors associated with the pathogenesis of *P. multocida* (Harper and Boyce, 2017). The LPS of *P. multocida* consists of a highly hydrophobic lipid A and inner and outer core oligosaccharide backbones, but lacks the typical extended O-antigen structure (Harper et al., 2011; Peng et al., 2019). Transcriptome sequencing results clarified that *lpxD* (lipid A biosynthesis), *VipA* (Vi polysaccharide biosynthesis protein), *galE* and *capD* (lipopolysaccharide biosynthetic process) were significantly down-regulated in PmCQ2Δ0442. Moreover, the LPS content was also sharply reduced. But the mechanism by which *Pm0442* affects LPS synthesis-related genes is still unclear.

The capsule and LPS of *P. multocida* constitute the major components of the bacterial cell surface, which play key roles in a range of interactions between the bacteria and the hosts. Both polysaccharides are involved in resistance to phagocytosis, they are therefore essential for virulence (Harper et al., 2006; Peng et al., 2019). PmCQ2Δ0442 associated with macrophage was significantly increased, indicating *Pm0442* affects capsule and LPS synthesis to affects adhesion and phagocytosis. As a result, the bacterial loads and pathological changes of mouse lung tissue caused by PmCQ2Δ0442 strongly decreased.

More than 2.5% (53 genes) of the total genes in the Pm70 genome encode proteins involved in iron uptake or acquisition (May et al., 2001). Similarly, more than 2.1% (46 genes) of all genes in the HB01 genome are expected to encode proteins specifically for iron uptake or acquisition (Peng et al., 2016). Genome-wide sequencing results indicated that more than 2.4% (53 genes) of the total genes in the PmCQ2 (GenBank: CP033599.1) are predicted to encode proteins for iron uptake or acquisition (Supplementary Table S3). We found that the expression of TonB receptor related genes (*hgpA*, *hemR*, *tdhA*, and *HI_1369*) and iron regulation/acquisition related genes (*FhuB/hmuV*, *SitA*, *SitB*, *SitC*, *SitD*, *tbpA*, *afuA*, *afuB*, *hutZ*, *chuX*, and *hugZ*) were markedly reduced in PmCQ2Δ0442 based on RNA sequencing. *TbpA* gene encodes transferrin-binding protein A that is important for iron uptake in *Pasteurellaceae* (Harper et al., 2006). The *afuABC* operon encodes the Afu iron transport system, which is similar to that found in *Actinobacillus pleuropneumoniae* (Xu et al., 2008). The *fecABCDE* operon encodes proteins involved in the synthesis of the TonB-dependent transporter (TBDT) system (Peng et al., 2016). The hemoglobin binding protein encoded by *hgbA* gene is homologous to that found in *A. pleuropneumoniae* (Srikumar et al., 2004). The proteins of *FhumB* and *FhumC* involve transporting free heme into cytoplasm, which are similar to the proteins found in *Haemophilus parasuis* (Xu et al., 2011). The *SitABCD* operon encodes the periplasmic iron transport system and is homologous to *yefABCD* found in *Mannheimia haemolytica* (Roehrig et al., 2007). Study has shown that iron deprivation significantly reduces the capsular content of *P. multocida* (Jacques et al., 1994), but the specific mechanism remains unclear. The result indicates that *Pm0442* is an important virulence gene, but the specific regulation mechanism of iron uptake needed.

Innate immune responses play important roles in host defense against bacterial infection. As an important component of the mammalian natural immune system, TLRs are expressed in immune cells such as macrophages, dendritic cells (DCs) and B lymphocytes (Brown et al., 2011). Our previous study found that TLR4 receptor mediates the production of pro-inflammatory cytokines in the macrophages activated by PmCQ2, but other receptors may also be involved in this process (Fang et al., 2019). This study found that the production of inflammatory cytokines was significantly reduced in TLR2^–/–^ macrophages compared to WT macrophage. This result indicates that TLR2 is also involved in the secretion of inflammatory factors in macrophages infected by PmCQ2.

Lipoproteins are important proteins on the surface of bacteria, and many bacterial lipoproteins act as inflammatory mediators to stimulate the host immune responses. TLR2 plays an important role in the recognition of bacterial lipoproteins even at a very low concentration (Zahringer et al., 2008). TLR2 recognizes lipoproteins in two ways, one of which is similar to TLR4 for identifying LPS, in the form of acylation heterodimers, and the recognition site is the N-terminal cysteine of lipoproteins. For instance, LprA of *Mycobacterium tuberculosis*, OspA of *Borrelia burgdorferi*, and U-OMP19 and U-OMP16 of *Brucella abortus* can activate TLR2 only in the presence of acylation (Morrison et al., 1997; Giambartolomei et al., 2004; Pecora et al., 2006). The other way is direct recognition without acylation, thereby activating the TLR2-MyD88 signaling pathway and regulating the immune responses. For example, the two lipoproteins of Rv0577 and LprG of *Mycobacterium tuberculosis* still have TLR2 activity in the absence of acylation (Drage et al., 2010; Byun et al., 2012). This is also true for MPT83 of *M. tuberculosis*, LipL32 of *Leptospira* and Tp17 of *Treponema pallidum*, indicating that TLR2 activity does not require acylation for certain lipoproteins (Hsu et al., 2010; Chen et al., 2012; Zhang et al., 2015). In this study, PM0442 induced macrophages to secrete IL-1β, TNF-α, IL-6, and IL-12p40 in a dose-dependent manner. Notably, antibody blocking of TLR2 and genetic deficiency of TLR2 show lower secretion of IL-1β, TNF-α, IL-6, and IL-12p40 after PM0442 treatment. Indeed, the acylation site of the *Pm0442* gene was removed in the experiment, and PM0442 protein interacts directly with TLR2 of macrophages *in vitro* by His-pull-down assay. Importantly, PmCQ2Δ0442 mutant decreases the production of inflammatory cytokine in macrophages depending on TLR2. However, the underlying mechanism for TLR2 to recognize PM0442 protein needs further investigations.

Priya et al. (2017) analyzed the recruitment of key signaling pathways in mice after *P. multocida* infection by gene chip method, and found that the differentially expressed genes are highly enriched in NF-κB and MAPK signaling pathways, which are consistent with our previously reported transcriptomic sequencing results of mice lung tissue infected by PmCQ2 (Wu et al., 2017). NF-κB is a multifunctional transcription factor, and participates in immune responses and inflammatory responses *in vivo* (Liu T. et al., 2017). NF-κB is inactivated by binding to IκB at rest and is mostly present in the cytosol. After activation, NF-κB transfers from the cytoplasm to the nucleus to induce transcription of genes, including those for IL-1β, TNF-α, IL-6, and IL-12p40 (Xiang et al., 2015; Fan et al., 2016). We found that PM0442 activates NF-κBp65 phosphorylation within 30 min and achieves this process by inducing rapid degradation of IκBα. Notably, NF-κB inhibitor BAY11-7082 can significantly inhibit the secretion of cytokines IL-1β, TNF-α, IL-6, and IL-12p40 induced by PM0442. This result indicates that the NF-κB signaling pathway has a significant effect on the secretion of the IL-1β, TNF-α, IL-6, and IL-12p40 in macrophages induced by PM0442.

MAPKs are protein kinases that are widely distributed in mammalian eukaryotic cells with a double phosphorylation capacity of serine and tyrosine, including p42/44 extracellular signal-regulated kinase ERK, c-Jun NH2-terminal kinase JNKs and p38 MAPK (Johnson and Lapadat, 2002). PM0442 protein activates the phosphorylation of ERK1/2 and p38 in downstream MAPK signaling pathway of TLR2, but has little effect on the phosphorylation of JNK. Further studies have shown that inhibitors of ERK1/2 and p38 (U0126 and SB203580) signaling pathways significantly inhibit the secretion of cytokines in macrophages, but not JNK inhibitor (SP600125). This result demonstrates that p38 and ERK1/2 MAPK signaling pathways play a significant role in secreting IL-1β, TNF-α, IL-6, and IL-12p40 in PM0442-induced macrophages. However, it is unknown whether other signaling pathways, like mTOR, are also involved in this process (Xia et al., 2018, 2019; Ren et al., 2019). Also, it is interesting to uncover the effect of PM0442 on the cellular metabolism as the metabolic reprogram highly shapes the fate decision of macrophages.

In conclusion, *Pm0442* affected capsular and LPS synthesis and iron uptake-related gene expression affecting adhesion and phagocytosis. Furthermore, PM0442 bound directly to Toll-like receptor 2 (TLR2) to mediate pro-inflammatory cytokine secretion of IL-1β, TNF-α, IL-6, and IL-12p40 in macrophages via activation of the NF-κB, ERK1/2 and p38 signaling pathways. Notably, PmCQ2Δ0442 is a potential vaccine candidate strain. Our findings demonstrate that *Pm0442* is a virulence-related gene that affects other virulence-related gene expression, and PM0442 protein directly binds to TLR2 to induce inflammatory responses of mice, which provides new guidance for the prevention and control of Pasteurellosis.

## Data Availability Statement

The datasets generated in this study can be found in online repositories. The names of the repository/repositories and accession number(s) can be found below: https://www.ncbi.nlm.nih.gov/, PRJNA597831.

## Ethics Statement

The animal study was reviewed and approved by the Laboratory Animal Welfare and Ethical Commission of the Southwest University (Permit No. 11-1025), Chongqing, China.

## Author Contributions

YP and NL designed the experiments. FH, XQ, NX, PL, XW, LD, and YD conducted the experiments. FH, XQ, RF, and NL analyzed the data and prepared the figures. FH, XQ, and PH drafted the manuscript. YP, RF, NL, and PH revised and approved the final manuscript. All authors contributed to the article and approved the submitted version.

## Conflict of Interest

The authors declare that the research was conducted in the absence of any commercial or financial relationships that could be construed as a potential conflict of interest.

## Supplementary Material

FIGURE S1 The transcription of amino acids, carbohydrate and energy metabolism, and membrane transport-related genes were changed *in vitro*. **(A)** PCA of PmCQ2 and PmCQ2Δ0442 *in vitro* (*n* = 3). **(B)** Heat map for clustering DEGs of PmCQ2 and PmCQ2Δ0442 *in vitro* (FC ≥ 2). **(C)** The up/down-regulated DEGs of PmCQ2 and PmCQ2Δ0442 *in vitro* (FC ≥ 2). **(D,E)** Classification of KGEE Pathway **(D)** and GO function classification **(E)** of PmCQ2 and PmCQ2Δ0442 *in vitro*. **(F,G)** Amino acid metabolism, carbohydrate and energy metabolism, and membrane transport related DEGs in RNA-seq **(F)** and in RT-PCR (*n* = 3) **(G)**. Panel **(G)** were representative of two independent experiments with 3 replicates per group and analyzed by multiple comparative analysis, and expressed as means ± SD (^∗^*p* < 0.05, ^∗∗^*p* < 0.01).

FIGURE S2 Capsular polysaccharide-related genes significantly down-regulated in PmCQ2Δ0442. **(A,B)** Capsular polysaccharide-related DEGs in RNA-seq **(A)** and in RT-PCR (*n* = 3) **(B)**. Panel **(B)** were pooled from two independent experiments with 3 replicates per group and analyzed with multiple *t*-test. The data were expressed as means ± SD. ^∗^*P* < 0.05, ^∗∗^*P* < 0.01.

FIGURE S3 PM0442 induces the secretion of inflammatory cytokines in RAW264.7 macrophages. **(A–D)** 10 μg/mL rPM0442 protein induces a large amount secretion of IL-1β **(A)**, TNF-α **(B)**, IL-6 **(C)**, and IL-12p40 **(D)** in RAW264.7. Data were pooled from three independent experiments with 6 replicates per group. Statistical significance was analyzed with one-way ANOVA and expressed as means ± SD (^∗∗∗^*P* < 0.001).

FIGURE S4 Pm0442 of PmCQ2 plays critical roles in secretion of cytokines in macrophages through TLR2. **(A–D)** Peritoneal macrophage from C57BL/6 WT, Tlr2^–/–^, and Tlr4^–/–^ mice were left uninfected (UI) or infected with PmCQ2, PmCQ2Δ0442, and PmCQ2Δ0442/pPm0442 at an MOI of 1, and then the supernatants were collected at 24 h post-infection. The levels of IL-1β **(A)**, TNF-α **(B)**, IL-6 **(C),** and IL-12p40 **(D)** in the supernatants were determined by ELISA. The data were pooled from two independent experiments with 8 replicates per group. Panels **(A–D)** were analyzed by multiple comparative analysis, and expressed as means ± SD (^∗^*p* < 0.05, ^∗∗^*p* < 0.01, ^∗∗∗^*p* < 0.001, ^****^*p* < 0.0001).

FIGURE S5 PmCQ2Δ0442 decreased the production of inflammatory cytokines in RAW264.7 macrophages. **(A–D)** PmCQ2Δ0442 reduces a large amount secretion of IL-1β **(A)**, TNF-α **(B)**, IL-6 **(C)**, and IL-12p40 **(D)** in RAW264.7. Data were pooled from three independent experiments with 6 replicates per group. Statistical significance was analyzed with one-way ANOVA and expressed as means ± SD (^∗^*p* < 0.05, ^∗∗^*p* < 0.01, ^∗∗∗^*P* < 0.001).

TABLE S1 Predicted 67 Lipoproteins of PmCQ2 based on whole genome sequencing.

TABLE S2 Significant up-regulated 37 lipoproteins of PmCQ2 based on transcriptome sequencing.

TABLE S3 Predicted 53 iron utilization related genes of PmCQ2 based on whole genome sequencing.

TABLE S4 qRT-PCR Primers used in this work.
