# Supplementary figures and images for: Pasteurella multocida Pm0442 Affects Virulence Gene Expression and Targets TLR2 to Induce Inflammatory Responses
Source: Front Microbiol. 2020 Aug 14;11:1972. doi: 10.3389/fmicb.2020.01972 (PMC7456837; doi:10.3389/fmicb.2020.01972)

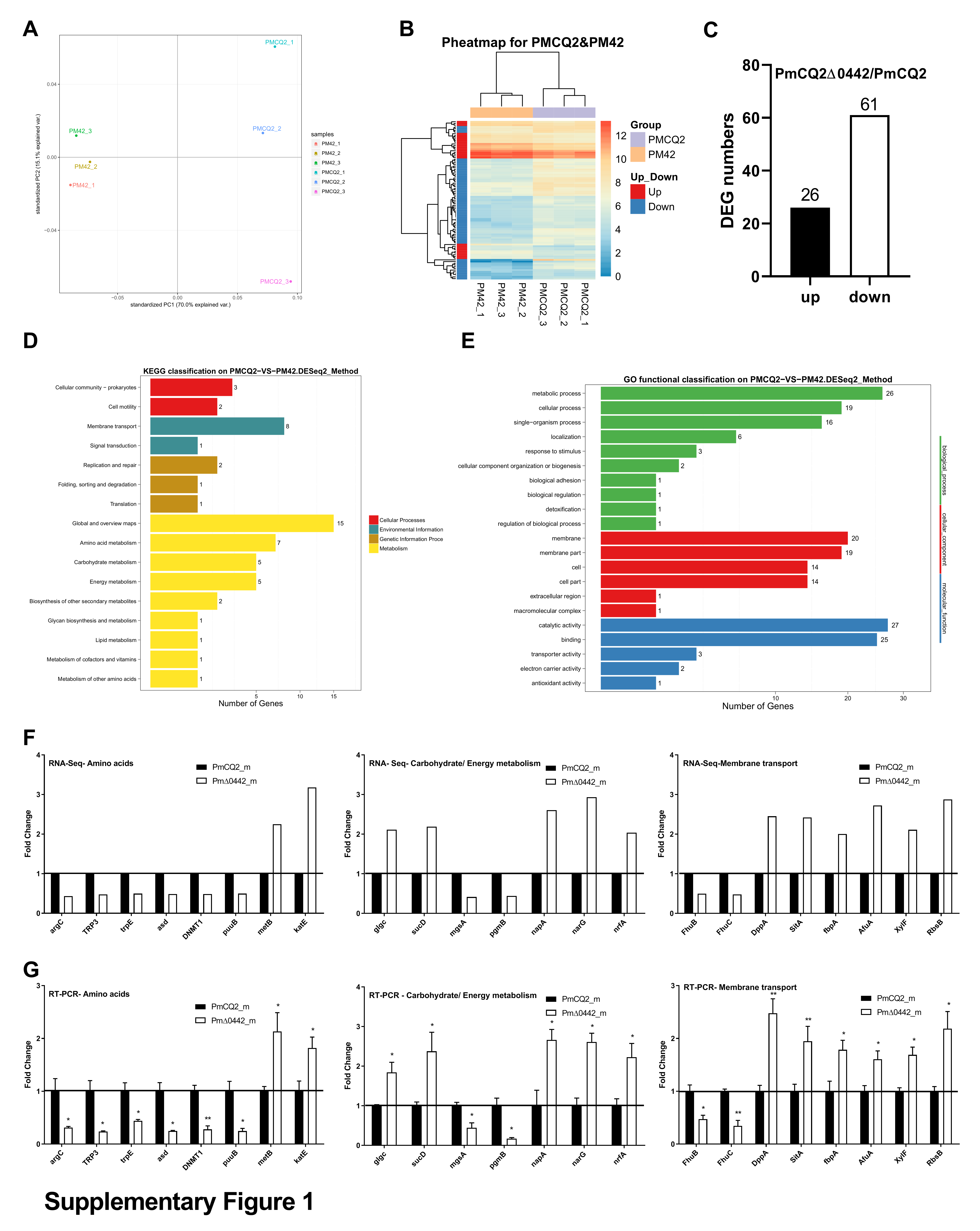

Supplement: FIGURE S1 — The transcription of amino acids, carbohydrate and energy metabolism, and membrane transport-related genes were changed in vitro. (A) PCA of PmCQ2 and PmCQ2Δ0442 in vitro (n = 3). (B) Heat map for clustering DEGs of PmCQ2 and PmCQ2Δ0442 in vitro (FC ≥ 2). (C) The up/down-regulated DEGs of PmCQ2 and PmCQ2Δ0442 in vitro (FC ≥ 2). (D,E) Classification of KGEE Pathway (D) and GO function classification (E) of PmCQ2 and PmCQ2Δ0442 in vitro. (F,G) Amino acid metabolism, carbohydrate and energy metabolism, and membrane transport related DEGs in RNA-seq (F) and in RT-PCR (n = 3) (G). Panel (G) were representative of two independent experiments with 3 replicates per group and analyzed by multiple comparative analysis, and expressed as means ± SD (∗p < 0.05, ∗∗p < 0.01). [file Image_1.TIF]
